# Mismatch Response to Polysyllabic Nonwords: A Neurophysiological Signature of Language Learning Capacity

**DOI:** 10.1371/journal.pone.0006270

**Published:** 2009-07-17

**Authors:** Johanna G. Barry, Mervyn J. Hardiman, Dorothy V. M. Bishop

**Affiliations:** 1 Department of Experimental Psychology, University of Oxford, Oxford, United Kingdom; 2 Max Planck Institute for Human Cognition and Brain Sciences, Leipzig, Germany; 3 MRC Institute of Hearing Research, Nottingham Clinical Section, Nottingham, United Kingdom; Victoria University of Wellington, New Zealand

## Abstract

**Background:**

The ability to repeat polysyllabic nonwords such as “blonterstaping” has frequently been shown to correlate with language learning ability but it is not clear why such a correlation should exist. Three alternative explanations have been offered, stated in terms of differences in: (a) perceptual ability; (b) efficiency of phonological loop functioning; (c) pre-existing vocabulary knowledge and/or articulatory skills. In the present study, we used event-related potentials to assess the contributions from these three factors to explaining individual variation in nonword repetition ability.

**Methodology/Principal Findings:**

59 adults who were subdivided according to whether they were good or poor nonword-repeaters participated. Electrophysiologically measured mismatch responses were recorded to changes in consonants as participants passively listened to a repeating four syllable CV-string. The consonant change could occur in one of four positions along the CV-string and we predicted that: (a) if nonword repetition depended purely on auditory discrimination ability, then reduced mismatch responses to all four consonant changes would be observed in the poor nonword-repeaters, (b) if it depended on encoding or decay of information in a capacity-limited phonological store, then a position specific decrease in mismatch response would be observed, (c) if neither cognitive capacity was involved, then the two groups of participants would provide equivalent mismatch responses. Consistent with our second hypothesis, a position specific difference located on the third syllable was observed in the late discriminative negativity (LDN) window (230–630 ms post-syllable onset).

**Conclusions/Significance:**

Our data thus confirm that people who are poorer at nonword repetition are less efficient in early processing of polysyllabic speech materials, but this impairment is not attributable to deficits in low level auditory discrimination. We conclude by discussing the significance of the observed relationship between LDN amplitude and nonword repetition ability and describe how this relatively little understood ERP component provides a biological window onto processes required for successful language learning.

## Introduction

Language dominates almost every aspect of human life, yet the biological basis for this central component of human functioning remains largely a matter of debate. The notion that there might be a single factor that determines language capacity is no longer seen as tenable [1], [2]. Rather, it is agreed that our language faculty develops out of a range of both general and more domain-specific underlying capacities [3]. The focus of the present research is on probing the functioning of one such cognitive component which is postulated to be intimately involved in language learning, namely, the phonological loop.

The notion of an ‘articulatory loop’ was introduced by Baddeley and Hitch [4] to refer to a core component of verbal short-term memory which they hypothesised functioned to enable people to retain unfamiliar sequences of phonological material for short periods of time. The component was subsequently renamed the ‘phonological loop’ to reflect the fact that the material is encoded in a speech-based form. The loop system essentially comprises two parts: a storage component – possibly analogous to auditory sensory memory [5] – and a rehearsal component which updates and refreshes information encoded in the storage component. In normal adult language, the phonological loop seems to play little role beyond helping to remember material such as telephone numbers over brief periods. However, in the context of language learning, numerous studies have shown a strong correlation between phonological loop capacity and vocabulary development in children, even when effects due to nonverbal IQ are partialed out [6]–[8]. Similar findings have also been reported for both adult and child second language learners [e.g., 9], [10]. Finally, there are numerous reports in the literature demonstrating an association between deficits in phonological loop functioning and language learning difficulties associated with certain developmental disorders such as dyslexia [e.g., 11]; specific language impairment (SLI) [e.g., 12], [13]; and autism [e.g., 14], [15]. As an interesting counterpoint to these disorders, children with William's syndrome, who have fairly intact language learning abilities, show no deficits in phonological loop functioning, despite significant intellectual impairments [e.g., 16].

Based on the clear association between phonological loop functioning and language learning, Baddeley, Gathercole, and Papagno [17] argued that the primary role for the phonological loop is as a language learning device. In their view, the loop promotes language learning by providing a short-term holding bay for unfamiliar sound patterns, such as new words, or novel morphological or syntactic forms, until they can be encoded into long-term memory. Baddeley et al. [17] left open the precise mechanism for how the phonological loop facilitated language learning with the implication being simply that the greater its capacity, the more efficient and accurate the encoding of information into long-term memory.

The functioning of the phonological loop is probed using either serial recall or nonword repetition tasks. The latter task, which involves immediately repeating nonsense words like ‘blonterstaping’, is considered to be particularly sensitive. Despite its apparent simplicity, the successful performance of the task requires the recruitment of a range of cognitive skills such as accurate auditory discrimination of the acoustic elements in the incoming stimulus; phonological processing and encoding into short-term phonological memory; reprocessing into an appropriate motor program; and finally overt articulation. In the case of children with SLI, Gathercole and Baddeley [13] could find no evidence that their deficits in nonword repetition ability derived from differences either in articulation or auditory discrimination and concluded that the children's difficulties in performing the task were primarily due to deficits in phonological short-term memory. This conclusion is still widely accepted, though some contribution from articulation has been noted [12], and deficits in auditory processing abilities have also been reported for some but not all children with SLI [e.g., 18]–[20]. It is still not clear however, which aspect of phonological loop function specifically determines individual differences in nonword repetition. Gathercole and Baddeley [13] proposed three processes which might be impaired in poor nonword-repeaters: (a) analysis and encoding of speech input; (b) storage capacity; (c) rate of fading of the sensory memory trace.

At this point, it is important to clarify our position vis-à-vis *discrimination* versus *perception* of speech sounds. Discrimination involves distinguishing acoustic cues to phoneme identity, and is regarded by Gathercole and Baddeley as intact in SLI on the basis of near-ceiling performance on repetition of two syllable nonwords. Perception entails encoding the incoming acoustic signal as a phonemic representation. In adults with mature phonological systems, this process will result in the initial sounds of [bi], [bu] and [ba] being encoded as the same phoneme, /b/, despite their acoustic differences. Problems with encoding could arise, for example, if a child had an immature phonological system and failed to analyse speech in terms of the small set of phonemic segments comprising his/her native phonology [21]. Problems specifically with the processing of longer nonwords might be indicative of limitations on the number of representations that could be stored, as suggested by Gathercole and Baddeley [13], or, as we shall discuss further below, could also arise if the process of extracting phonemic representations was unable to keep pace with incoming auditory information.

Given its clear association with language learning, it is of considerable interest to understand what impacts on the successful functioning of the phonological loop. Such information would potentially provide a window into understanding why some people are better at second language learning or why some children demonstrate significant delays relative to their peers in acquiring their first language. The findings from the few studies available, attempting to explain the relationship between phonological loop functioning and word learning, demonstrate how controversial this question still is. Hartley and Houghton [22], for example, using a connectionist model of short-term memory showed how rapid fading of the sensory memory trace successfully explained the empirical data from typically-developing young children and two adults with short-term memory deficits. Čeponienė, Service, Sanna, Cheour, and Näätänen [23] combined behavioural and electrophysiological techniques to assess the role of sensory memory trace durability in explaining differences in nonword repetition ability in young children. The data were equivocal regarding the role of memory trace durability, but evidence was found for poorer processing by the poor nonword-repeaters of acoustically subtle differences in stimuli. More recently, Service, Maury, and Luotoniemi [24] assessed nonword learning by good and poor nonword-repeaters, using stimuli that contained redundant syllables. They demonstrated a benefit for syllable redundancy in good nonword-repeaters only and concluded that individual differences both in immediate recall and in cumulative learning from repeated exposures to novel materials were explained by differences in long-term phonological learning of the structure of novel phonological items.

In the present research, like Čeponienė et al. [23], we used electrophysiological rather than behavioural techniques to test directly for differences in early speech encoding and to observe indirectly effects resulting from differences in phonological loop functioning in good and poor nonword-repeaters. These latter effects were predicted to have a cumulative effect across long multisyllabic stimuli on the efficiency of early consonant change detection. We employed a procedure based on the passive elicitation of mismatch responses very early after auditory input i.e., the participant does not generate a verbal response or indeed attend to the stimulus. The mismatch response thus reflects automatic detection by the brain of a change in stimulus (the so-called deviant) after a memory trace has previously been established to a frequently heard standard stimulus. Because active involvement of the participant is explicitly excluded, the measures obtained are relatively free from many of the confounding factors typically associated with procedures involving a behavioural response.

Two kinds of mismatch response have been reported in the literature: the mismatch negativity (MMN) [25], which has been extensively studied, and the much less commonly reported late discriminative negativity (LDN) [26]. Both components are typically elicited at fronto-central electrodes in response to a stimulus which does not match a previously established sensory trace for a standard stimulus. At its most basic, the MMN is thought to index auditory discrimination. For example, during frequency discrimination of simple tones, the size of the MMN increases with increasing frequency difference between standard and deviant [25]. Furthermore, some studies have reported correlations between the size of the MMN and individual differences in frequency discrimination ability [27].

Relative to the MMN, the LDN (also referred to as the ‘late mismatch negativity’) has not been much studied. It was initially thought to be a speech-specific component involved in the automatic detection of differences in lexicality [26]. However, it has since been observed in response to a range of stimuli including sinusoidal [28] and complex tones [29]. Čeponienė, et al. [30] argued that since the relationship between the MMN and LDN latencies remains relatively fixed, the two components must reflect linked stages in the processing of change detection. In their view the MMN results from a purely sensory response to stimulus change, while the LDN, though still pre-attentive, occurs too late to be purely sensory and must therefore reflect a higher-order, i.e., a cognitive level, of stimulus processing. It remains unclear however, what exactly this higher level of processing is. Hill, McArthur, and Bishop [31] suggested the LDN indicated the recruitment of extra cortical structures for the processing of phonologically significant differences between stimuli, while Zachau, Rinker, Korner, Kohls, et al. [32] argued that it reflected the establishment of more permanent internal representations of recurring regularities in the incoming auditory stream. In general, views on the distinction between MMN and LDN appear to correspond to the distinction (noted above) between discrimination and extraction of a perceptual representation.

The stimuli employed in the present study were selected to mimic the nonsense materials used in the nonword repetition task. The standard stimulus was a four syllable long CV-string e.g., ‘ba-bi-bu-be’, against which four deviant stimuli were contrasted. The deviant stimuli differed from the standard in having a single consonant change from [b] to [d], which could occur at one of four possible positions along the CV-string e.g., ‘**d**a-bi-bu-be’ or ‘ba-bi-**d**u-be’. As a measure of auditory discrimination, the MMN permitted us to test the hypothesis that poor nonword repetition is related to problems at the earliest stage of encoding, i.e., in distinguishing between consonants. We predicted that if poor nonword repetition derived from deficits in discriminating the consonants in the auditory input, then a significant group difference would be observed due to reduced MMN responses to all four deviants by the poor nonword-repeaters.

It is known however, that differences in nonword repetition ability only begin to emerge with longer nonwords, hence the hypothesis of a capacity limited storage component in verbal short-term memory. We therefore designed our stimuli to test the effects of capacity limitations on information-processing in poor nonword-repeaters. We predicted that, if some aspect of storage capacity were impaired in the poor nonword-repeaters, the later syllables in the standard would be less well represented than the earlier syllables. Hence the process of change detection would be accurate only for the initial syllables. This would result in either, a progressive reduction of mismatch response which would be most pronounced for later syllables, or, a bow-shaped response pattern reflecting the sorts of primacy and recency effects reported in behaviorally-performed serial recall tasks. Thus when comparing good versus poor nonword-repeaters, we predicted a Group × Deviant interaction that could emerge either in the MMN or LDN, with the two groups showing similar performance for early syllables, but diverging for later syllables.

More recent theories of verbal working memory have proposed an important contribution from processes involved in serial ordering of incoming auditory materials [33]. In our paradigm, the same consonant appeared in all four syllables and the order of vowels was held constant for both standard and deviant stimuli in a block, meaning minimal demands were placed on the encoding of serial order. We therefore would not predict any impact on mismatch responses due to poorer serial ordering in the poor nonword-repeaters, if this is were the explanation for their deficits in nonword repetition.

Finally, it has been argued that differences in nonword repetition ability derive from factors quite external to a putative phonological loop: factors such as pre-existing differences in vocabulary, knowledge of phonotactic probabilities or differences in speech production ability [e.g., 34]–[36]. Since the mismatch response is recorded very early after auditory input and neither articulation or vocabulary knowledge play any role in the task, our paradigm thus permitted us to assess the degree to which such external factors determine individual differences in nonword repetition. Specifically, we predicted that if differences in nonword repetition derived primarily from constraints in articulatory output, then mismatch responses to all deviants would be equivalent across the two groups of participants. Similarly, vocabulary knowledge can facilitate nonword repetition performance through top-down influence where the nonwords resemble real words or where the component phonotactic patterns of the real word can facilitate repetition performance. However, such top-down effects are implausible in the context of the present paradigm design, since the repeated ‘ba-bi-bu-be’ standard syllable used here is not word-like, and rapidly becomes familiar to all participants in the course of the study. In sum, if we find group differences in mismatch responses to nonwords in this electrophysiological paradigm, they can only be attributed to abnormalities in the basic processes involved in the phonological loop, rather than being secondary to top-down influences due to differences in vocabulary or speech production skills.

## Results

**Figure 1:**
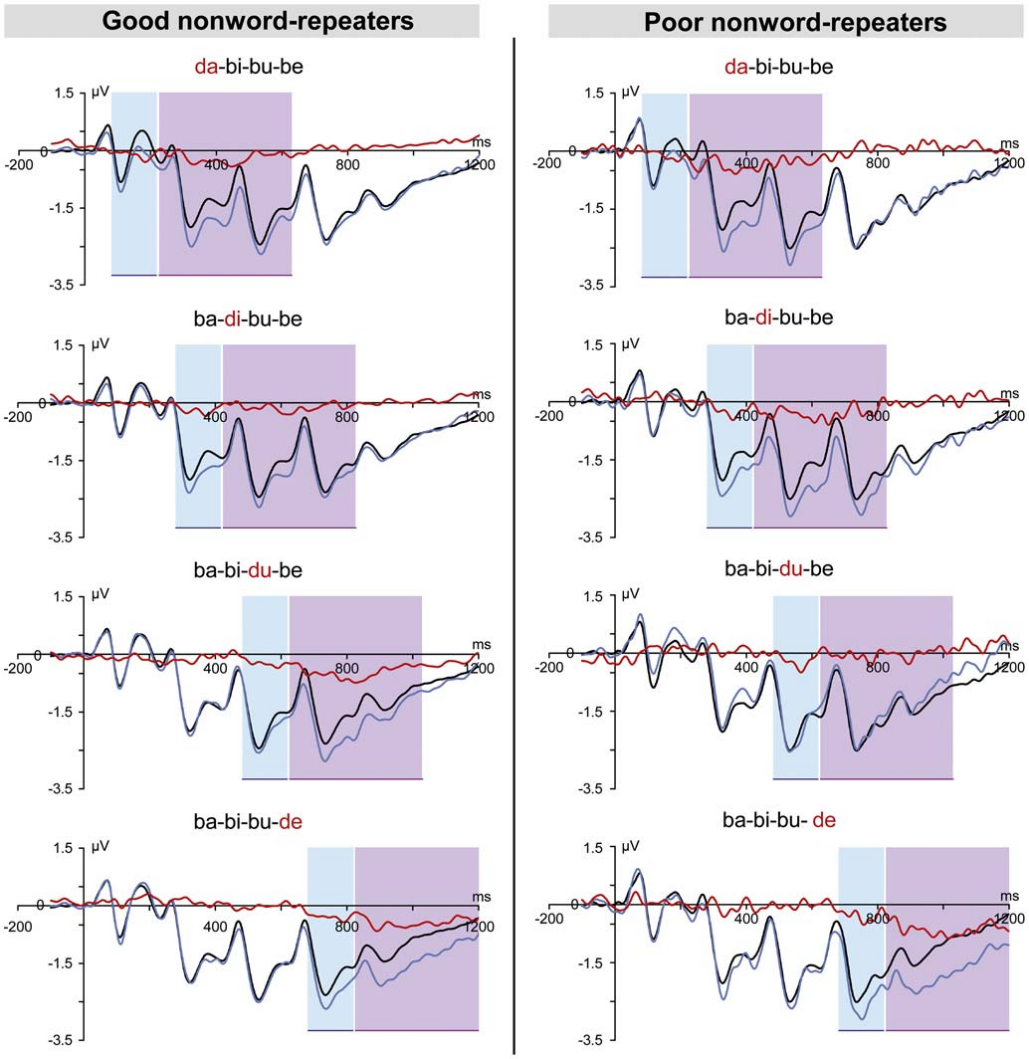
Comparison of grand-averaged ERPS at FZ for the four deviants in relation to the standard. Plots of time (ms) vs ERP amplitude (µv) for standard (thin blue line) and deviant (bold line) stimuli in relation to position of the deviant syllable as recorded at FZ, for good (left-hand panel) and poor (right-hand panel) nonword-repeaters. The raw difference waveform is shown in red. Each plot is labelled according to the location of the deviant consonant change relative to the standard e.g., ‘ba-bi-bu-be’. The shaded blue area denotes the interval over which the MMN was measured and the pink area the interval for the LDN.

The ERP responses at FZ to the standard and deviant stimuli, together with the resultant difference waves are plotted in Figure 1 for each participant group.

### Analysis of MMN amplitude and latency

As a first analysis, we compared the amplitudes of the MMN for the two groups of nonword-repeaters across the four deviants (Electrode × Deviant × Group). A significant main effect was found for Electrode [F(5, 53) = 4.807, p = .001, η^2^ = 0.312], but there was no main effect for Group nor did any interaction with Group approach significance. Thus the two groups did not differ in early consonant change detection.

In a similar analysis, peak latencies for the MMN responses were also compared between the groups. No significant main effects or interactions were obtained, i.e., the two groups did not differ in rate of processing during early consonant change discrimination.

### Analysis of LDN amplitude and latency

**Figure 2:**
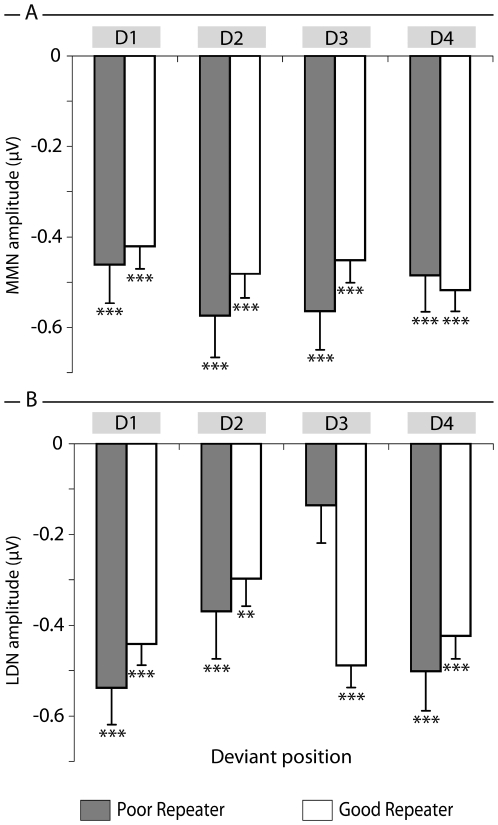
Comparison of mean MMN and LDN amplitudes for the six fronto-central electrodes. Mean amplitude of MMN (top panel) and LDN (lower panel) of the six analysed electrodes in relation to nonword repetition status and position of deviant syllable. Error bars show standard errors. Asterisks on the upper x-axis denote where the mean mismatch response differed significantly from zero on t-test, at p-values of .01 (**) or .001 (***).

Figure 2 compares mean LDN and MMN amplitudes collapsed across the six electrodes for the two groups as a function of deviant. As illustrated, strong LDN responses were obtained to all four deviants in the good nonword-repeaters. By contrast, an attenuated LDN response to D3 was observed in the poor nonword-repeaters.

Statistical analyses showed that, in addition to a significant main effect for Electrode [F(5, 53) = 5.725, p<.001; η^2^ = 0.351], there was a significant main effect for Deviant [F(3, 53) 3.629, p<.01; η^2^ = 0.165] and importantly, a significant Group × Deviant interaction [F(3, 55) 6.9, p<.01, η^2^ = 0.273]. The analysis was repeated with the good nonword-repeaters subdivided into three groups of 14–15 participants each, and the interaction with deviant position was replicated, F (9, 165) = 2.67, p = .006. The interaction was further explored by repeating the within-subjects analysis for good and poor nonword-repeaters separately. For the poor nonword-repeaters, there was a significant effect of deviant position, F (3,12) = 7.7, p = .004, η^2^ = 0.657, and specific contrasts revealed this reflected a significant quadratic term, F (1, 14) = 8.2, p = .013, η^2^ = 0.368, reflecting the bow-shaped function seen in the LDN. For the good nonword-repeaters, there was also a significant effect of deviant position, F (3, 41) = 3.6, p = .022, η^2^ = 0.207, but neither linear nor quadratic terms were significant (F-ratios<1).

This analysis was followed up with one sample t-tests on average mismatch responses across the six fronto-central electrodes to determine whether the LDN differed significantly from zero. For the good nonword-repeaters, a significant LDN was found at all syllable positions (i.e., for positions 1, 3, and 4, p<.001; for position 2, p<.01). By contrast, for the poor nonword-repeaters, LDN responses significantly greater than zero were recorded at positions 1, 2, and 4 (with p values of <.001), but not at position 3. In sum, there is a significant difference in magnitude of LDN response to consonant change between the two groups at the third syllable and this difference associates with differences in overall nonword repetition score.

To further test this association, we performed a correlation between nonword repetition score and mean LDN amplitude in response to the four deviants. A strong correlation was observed between nonword repetition score and mean LDN amplitude at syllable 3 only (r = −0.407, p<.001), i.e., smaller LDN amplitudes at this position were associated with lower nonword repetition scores.

As with the MMN response, peak latencies were submitted to analysis to test for differences in rate of processing deviance detection between the two groups. Though a significant effect for Electrode was obtained [F(3, 55) 3.404, p = .01, η^2^ = 0.243], there was no main effect for Group nor any interaction with Group suggesting similar rates of processing among the two groups during this stage of consonant change detection.

### Relationship of MMN to LDN

The LDN and the MMN are both elicited in response to a change in stimulus, yet only differences in LDN were associated with nonword repetition ability. A question thus arises regarding the extent to which these two negative deflections provide different information about the process of change discrimination in this paradigm. To assess this, mean MMN and LDN amplitudes across the six electrodes were calculated and a series of one-tailed Pearson product moment correlations were performed between the amplitudes of the two components for each deviant. We predicted a direct relationship between the two components if they reflected common processes.

Correlations between the LDN and the MMN amplitudes in response to deviant syllables 1, 2 and 3 fell far short of significance (r = −.18, −21, .00 respectively) when all participants were included in the analysis. The correlation between MMN and LDN for deviant syllable 4 when both groups were included was .34 (p = .009), which was significantly different from zero, even after Bonferroni correction (critical p-value of .012).

Correlations between the LDN and MMN amplitudes were also tested for each group separately applying the Bonferroni corrected critical p-value of 0.012. No evidence was found for significant correlations between LDN and MMN in the poor nonword-repeaters. In the good nonword-repeaters, weak correlations were observed between LDN and MMN at syllables 1 and 4 (r = .37, .32 respectively) which did not survive correction for multiple testing.

Overall, with the possible exception of the final syllable, the evidence for common processes being involved in the generation of the MMN and LDN responses was not compelling.

### Family history and nonword repetition ability

**Figure 3:**
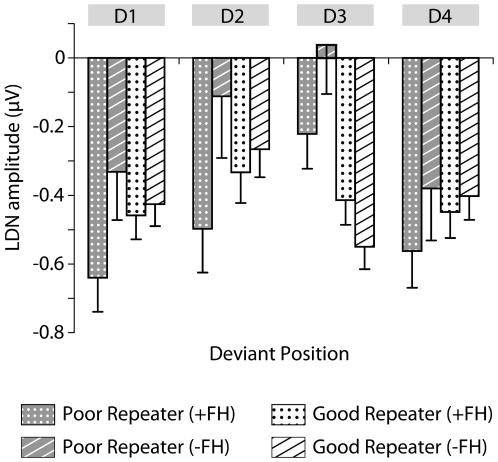
LDN amplitudes as a function of family history and nonword repetition status. Mean amplitude of LDN in relation to position of deviant syllable; nonword repetition status (Poor or Good); and, family history (+FH or −FH). Error bars show standard errors.

There were more parents of children with SLI in the poor nonword-repeater group. This raises a question regarding the role of family history for SLI in our findings. To test this, LDN amplitude × Deviant was entered into a repeated measures analysis with Group × Family history (+FH, −FH). The numbers are not sufficient for a powerful analysis, but no significant interaction was observed between family history and deviant position. A plot of the mean LDN amplitudes for each of the four groups (Figure 3) clearly demonstrates a reduction in LDN amplitude in response to the consonant change which, regardless of family history, occurs on the third syllable in the poor nonword-repeaters.

## Discussion

There is considerable controversy regarding what exactly the nonword repetition task is tapping into that makes it such a good predictor of language learning. In this study, we employed stimuli that were designed to explore the functioning of a hypothesized phonological storage system. We exploited the electrophysiologically-measured mismatch response to test for sensitivity to change at different syllable positions in good and poor nonword-repeaters. We predicted that if nonword repetition ability was determined by factors ancillary to the phonological loop, then depending on which factor was primary, we would see either:

(a) Significantly reduced MMN responses for poor nonword-repeaters to the deviants at all four syllable positions which would point to deficits in early auditory discrimination; or

(b) No group or syllable-position effect if differences in nonword repetition derive from factors extrinsic to the phonological loop, such as differences in motor ability or in vocabulary knowledge.

Alternatively, if a syllable-specific group difference emerged, this would suggest that factors associated with a capacity-limited storage system were impacting on the efficiency of information processing and change detection.

In the context of our three predictions, two findings are particularly noteworthy. First, the two groups of nonword-repeaters had similar early consonant change discrimination abilities as indicated by the MMN response i.e., accuracy of early encoding of incoming auditory information was similar between the groups. In the context of children with SLI, Gathercole and Baddeley [13] first argued that deficits in nonword repetition ability could not be wholly attributed to differences in speech discrimination ability. Our findings with adults with poor nonword repetition skills are consistent with that view. Second, in the poor nonword-repeaters, the LDN response was abolished for the consonant change occurring at the third syllable of the CV-string, resulting in a significant Group × Deviant interaction. As noted above, this pattern of results is not consistent with the idea that the group difference on behavioral tests of nonword repetition is explicable solely in terms of factors such as pre-existing vocabulary knowledge or differences in articulation skills.

Rather different conclusions were reached by Čeponienė et al. [23], who compared responses to deviant changes using a similar paradigm to ours to investigate the discrimination of just noticeable differences in two nonsense syllables ‘ba-ka’ versus ‘ba-ga’ in young good and poor nonword-repeaters. Contrary to our own conclusions with adult participants, their data suggest a role for discrimination deficits in young poor nonword-repeaters. However, their stimuli, unlike ours, were difficult to discriminate by design and shorter in length. Moreover their participants were younger. It is likely that the different conclusions arrived at by Čeponienė et al. reflect a range of factors including the subtlety of the acoustic differences to be discriminated and maturational differences between the participants in the two studies.

Gathercole and Baddeley [13] suggested three possible candidates directly associated with the phonological loop as impacting on its function: quality of initial encoding into the loop; storage capacity; and the rate of fading of the memory trace once encoded there.

At first glance, poor encoding seems inadequate to explain the results, because of the intact MMN responses seen in poor nonword-repeaters at all syllable positions, indicating adequacy of the early stages of speech discrimination. A simple storage account is also hard to reconcile with the results. If, for instance, poor nonword-repeaters could retain few syllables in memory, we might expect to see more pronounced deficits in their discriminative responses for both the third and fourth syllables, whereas the LDN attenuation was found for the third syllable only.

The notion of a rapidly fading memory trace has been proposed to explain deficits in verbal working memory [22], [37], but seems implausible to account for our results for two reasons. First, in an earlier task, using pure tone stimuli with variable inter-stimulus intervals, Barry, Hardiman, Line, White, Yasin, and Bishop [38] showed that, although parents of children with SLI have less durable sensory memory traces than parents of typically-developing children, these differences did not associate with differences in nonword repetition ability. Second, in this paradigm, the temporal gap (and hence opportunity for decay) was held constant between each standard syllable and its deviant analogue. If it was only rate of memory trace decay that distinguished the two groups of participants, one would not predict the observed syllable-specific position effect that was observed here.

It seems then that whatever differentiates good from poor nonword-repeaters is associated with information-processing under conditions of high input load. The position-specific group differences were not reflected in the early MMN response. They only became apparent in the LDN response. This, together with a lack of correlation between the two mismatch response types, suggests that the LDN provides different information about the processing of the auditory input. In the light of previous research, we suggest that the LDN is an index of formation of a phonological representation. This corresponds to a process of encoding into short-term memory, giving a more robust representation that can persist long enough to allow comparison between deviant and standard nonwords.

Why should this encoding process be selectively impaired for the third syllable of a four syllable nonword? One interpretation is in terms of the demands the task places on rapid processing of sequential information. The notion of differences in rate of processing of incoming auditory information derives from the SLI literature, where it has been hypothesized that ability to rapidly process incoming auditory stimuli is deficient in people affected by a language or literacy disorder [39]. The results from both behavioral and electrophysiological studies probing the validity of this hypothesis have been fairly mixed, but in a meta-analysis of studies investigating mismatch responses to syllables in children with language or literacy problems, Bishop [40] concluded that deficits in auditory processing were more likely to be observed when stimuli were presented in rapid succession.

Previous MMN studies in populations with language impairments have mostly focused on single syllables, but it may be that deficits in rate of processing only become apparent when processing multiple syllables. This hypothesis was tested by Kujala, Halmetoja, Näätänen, Alku, Lyytinen, et al. [41] who assessed the ability of participants with dyslexia to discriminate changes in vowel durations embedded in three syllable CV-strings (e.g., ‘ta-ta-ta’ versus ‘ta-taa-ta’). They observed no deficits pre-attentively to the change in vowel duration. However, when the participants were required to attend to the stimuli, they were less accurate at locating the change in syllable duration and they showed a significantly reduced N2b component in response to duration changes in the final syllable of the CV-string. These findings are somewhat reminiscent of our own findings. As such they are of interest given the overlap between dyslexia and SLI and given the fact that deficits in nonword repetition have been reported for both disorders [42].

Within the context of the behavioral literature on SLI, Gathercole [43] observed that nonword repetition by language-impaired children was more accurate when nonsense syllables were presented singly with short intervals between them, than when they were presented in a string. Again this supports the notion that ability to rapidly process incoming sequences of stimuli embedded within other complex stimuli plays an important role in nonword repetition and hence in language learning.

One problem for this account of findings is that one might expect to see effects on peak latencies of MMN and/or LDN, reflecting the slower rate of syllable-processing in the poor nonword-repeaters. This was not observed. Nevertheless, in other regards, the hypothesis makes sense of the specific pattern of results obtained here. A slower rate of processing of incoming speech would have a cumulative effect across the CV-string up to the final syllable where, because there is no subsequent syllable, perceptual analysis could be completed with a consequent recovery in LDN amplitude. In effect, this is an explanation in terms of deficits in encoding. It maintains that despite adequate early discrimination processes, poor nonword-repeaters fail to generate a robust phonological representation memory. These problems however, only become evident at rapid rates of stimulus presentation.

A radically different type of encoding account is suggested by the literature on perceptual grouping. Horváth, Czigler, Sussman, & Winkler [44] demonstrated that mismatch responses can be elicited by both global and local features of stimuli. In our design, we treated the global stimulus ‘ba-bi-bu-be’ as the standard to be compared with deviants differing on one syllable. However, this stimulus contains within it a local repeated phoneme, /b/, which potentially acts as a standard. Thus on hearing ‘ba-bi-**d**u-be’ the response to the third syllable might be influenced both by the deviance from the standard, but also by the deviant /d/ after a train of preceding /b/ consonants, both within the same syllable and from the preceding nonwords. Limited ability to segment individual phonemes has been mooted as a possible cause of poor nonword repetition [21], raising the possibility that poor nonword-repeaters fail to engage in local processing and so are influenced solely by global mismatch. Although this explanation fits well with prior theoretical speculations about nonword repetition, it does not readily account for the pattern of results obtained here, because when progressing through the four syllables of the nonword, one would expect to see a steadily increasing impact of local mismatch, as the number of prior standards increases. We cannot rule out the possibility that such a process contributes to the profile of results obtained here, but it would need further testing with materials designed to evaluate this explanation. If local processing is involved in mismatch generation only in good nonword-repeaters, then a clear prediction is that mismatch responses will be reduced (and resemble those seen in poor nonword-repeaters) if a different consonant were used for each syllable of the standard.

Overall, the results from this study suggest a link between two different theoretical accounts of factors affecting language learning. The auditory temporal processing account of Tallal [45] has a long history, but evidence has been mixed [46]. Most empirical studies have considered discrimination of pairs of tones or speech sounds, whereas the current study would suggest that, as the impact of slow processing is cumulative so that longer sequences of sounds are needed to reveal a deficit. The notion that phonological short-term memory is important for language-learning also has a long history. Within the context of these this theory, but the focus has been on explaining poor nonword repetition in terms of storage limitations or of rapid decay of representations. Encoding explanations have tended to be dismissed on the grounds that such problems should be apparent in short nonwords with one- or two-syllables. We suggest that this view is mistaken, because encoding is affected by the presence of adjacent syllables, and so will become apparent only in the later syllables of longer nonwords. The problem of poor nonword-repeaters seems to reflect an inability of encoding mechanisms to keep pace with incoming input. This would explain why nonword repetition is a more sensitive index of language difficulties than more conventional verbal memory span tasks, which typically adopt a slower rate of presentation.

Though the focus of this study has been on factors affecting nonword repetition, our participants were heterogeneous with respect to the language learning status of their child. The poor nonword-repeater group included a sizable minority of parents of typically-developing children, just as the good nonword-repeater group included many parents of children with SLI. However, as a further analysis of the data showed (Figure 3), the effects reported here are specific to nonword repetition ability and not necessarily associated with having a language impairment child *per se*. One must therefore ask, given the composition of our participant groups, what implications do our findings have for current understanding of the etiology of developmental disorders such as dyslexia and SLI?

Much of the research published to date has focused on finding a single underlying cause for a developmental language disorder, but it is becoming increasingly clear that a deficit in any one single underlying cognitive skill is unlikely to explain the broad range of phenotypes captured under simple umbrella terms such as SLI or dyslexia. Instead as Bishop [47] has argued, these disorders are more likely to develop out of a confluence of risk and protective factors, some of which are heritable. In the context of this study, deficits in the ability to process rapidly presented incoming auditory input seem to represent one such risk factor.

In sum, previous suggestions for language specialization in the human brain have focused mainly on categorical speech perception, speech production, processing of serial order, and syntactic processing [1]. Our data suggest that human language learning capacity is boosted by being able to process sequentially-presented verbal material rapidly enough to permit the accurate recognition of syllables occurring at the rate of 5 per second, without earlier syllables interfering with the processing of later ones. We conclude that without this ability, it is hard to learn polysyllabic words or to discriminate the non-redundant information contained within a rapidly changing speech stream.

## Materials and Methods

### Ethics statement

The study was approved by the Oxford Psychiatric Research Ethics Committee (OPREC) and informed signed consent was obtained for all participants.

### Subjects

Fifty-nine adults aged between 33 and 56 years were recruited from a previous study of parents of children with Specific Language Impairment (SLI) and parents of typically-developing children [48]. Unlike parents of typically-developing children, parents of children with SLI tend to be poor at nonword repetition even if unaffected by the disorder [48]. We thus had access to a sufficiently large number of participants with deficits in the task. All participants were native speakers of English, had normal hearing (bilateral pure tone test at 25 dB HL ISO for 500, 1000, and 2000 Hz), had an estimated nonverbal IQ of greater than 85 WASI (Wechsler Abbreviated Scale of Intelligence) [49], and no frank neurological damage. Nonword repetition was assessed using a subtest from the developmental neuropsychological assessment test battery (NEPSY) [50] which comprises 13 nonwords varying from two to five syllables in length. Scores were based on the number of correct syllables (maximum 46). The mean score for parents of typically-developing children was 40.9, s.d. 4.0; interquartile range 37–44. Participants were subdivided into two groups: good nonword-repeaters ≥37, (n = 44; 20 parents of children with SLI); or poor nonword-repeaters <37 (n = 15; 10 parents of children with SLI). This cutoff represents performance below the 10^th^ percentile in this group which we defined as clinically significant. The two participant groups were well matched for age, nonverbal IQ and sex (Table 1). Nonetheless, as would be expected given the relationship between nonword repetition and language learning, the poor nonword-repeaters completed fewer years of education and had more evidence of language and literacy problems, as assessed by an in-house spelling test, and two subtests from the Test of Word Reading Efficiency (TOWRE) [51]. There was also a non-significant trend for poorer performance on the Test for Reception of Grammar-2 (TROG-2), [52].

**Table 1:** Participant characteristics in relation to nonword repetition status such that ‘Good repeater’ refers to people with scores ≥37 and ‘Poor repeater’ refers to scores <37.

	Good Repeaters	Poor Repeaters	Effect size (η^2^)	p-value
	N = 44	N = 15		
Age	43.41 (5.29)	44.21 (6.51)	−.13	.634
Male:female	7:37	4:11	n/a	.356
Age left full-time education	19.64 (2.75)	16.79 (2.12)	1.16	.001
WASI Non-verbal IQ	112.50 (12.61)	112.73 (12.98)	−.01	.951
Digit repetition raw	10.59 (2.08)	9.27 (1.94)	.66	.035
Word reading scaled	93.95 (12.14)	83.93 (14.99)	.73	.012
Non-word reading scaled	100.41 (12.66)	86.27 (14.23)	1.05	.001
TROG scaled	101.74 (7.02)	97.47 (9.72)	.50	.072
Nonword repetition	41.0 (2.79)	33.3 (3.6)	n/a*	

* Groups selected on this variable: no overlap in scores.

### ERP Procedure

**Figure 4:**
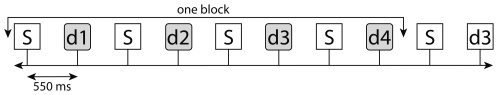
Summary of stimulus presentation for one block of standard and deviant stimuli. Each set of stimuli comprises one standard e.g., ‘ba-bi-bu-be’ and its corresponding four deviants e.g., d1 = ‘da-bi-bu-be’, d2 = ‘ba-di-bu-be’, d3 = ‘ba-bi-du-be’, d4 = ‘ba-bi-bu-de’. A total of 8 stimuli are presented per block i.e., four standards alternating with four equiprobable randomly ordered deviant stimuli.

Our stimulus presentation was based on the ‘Optimal MMN paradigm’ [53] which permits the rapid assessment of pre-attentive auditory discrimination of up to five different stimuli. A memory trace for the standard stimulus (e.g., ‘ba-bi-bu-be’) was first established by presenting a stream of 15 stimuli. This was followed by a series of blocks of four standard stimuli alternating in a quasi-random sequence with four equiprobable deviant stimuli (see Figure 4).

### Stimuli

The four syllable CV-strings used here were designed to be simplified analogues of the polysyllabic nonsense words used in the nonword repetition task. The same consonant was used in all syllables to minimize variation in the waveforms elicited by each syllable. Deviant stimuli involved a single change in consonant from [b] to [d] in one of the four CV elements along the string. Thus, where the standard was ‘ba-bi-bu-be’ the corresponding four deviants were: D1 = ‘**d**a-bi-bu-be’; D2 = ‘ba-**d**i-bu-be’; D3 = ‘ba-bi-**d**u-be’; D4 = ‘ba-bi-bu-**d**e’. To control for effects due to the change of vowel across the string, we used four sets of stimuli, each composed of one standard and four deviants. The four possible standards were: ‘ba-bi-bu-be’; ‘be-ba-bi-bu’; ‘bu-be-ba-bi’; and ‘bi-bu-be-ba’. Time constraints meant that it was only possible to present three sets of stimuli within a session. Participants were therefore presented with a random selection of three of out of these four possible sets to achieve counterbalancing of conditions across participants. Each set was presented as a separate block. Short pauses were provided between each set as and when required by the participant.

The CV-elements used to make up the strings were recorded using a standard female voice. Stimulus duration was 200 ms with a 20 ms roll off. Consonant burst differences were minimized and F0 was matched across the four syllables using Praat [54]. Finally, the amplitudes of the CV-elements were equalized before being combined into strings. The CV-strings were 800 ms in length and an inter-stimulus interval (ISI) of 550 ms was used. The presentation of one set of stimuli comprising a single standard and its four corresponding deviants lasted 13 minutes. A whole experiment involving three different sets of standards and deviants lasted up to 40 minutes.

### Experimental set-up

Participants were seated in an upright comfortable chair in a sound-attenuated, electrically-shielded booth and were requested to ignore the stimuli and keep movements to a minimum. To help them do this, they watched a silent video of their own choosing. Stimuli were presented monaurally to the right ear at 71 dB SPL through Sennheiser HD25-1 headphones.

### EEG recording and data analysis

The EEG was recorded on either a SynAmps (n = 7) or NuAmps NeuroScan Inc. system (n = 52) using Ag/AgCl sintered electrodes and a water-soluble conductive paste. Pilot studies indicated no system-related differences in ERPs measured and a subsequent repeated-measures ANOVA, with responses to D1 as the within-subjects measure, and System as the between-subjects measure, indicated no system-related differences in responses [F(1, 57) = 0.870, p = 0.102]. Choice of system was determined by availability and was not related to nonword repetition status of participants.

Subjects were fitted with either a Quick cap (n = 10) or an Easy cap (n = 49) with 31 electrodes approximating the 10–20 International system. Bipolar Electrooculograms were recorded from supra- and infra-orbital electrodes located around the left eye and also lateral to both the left and right eyes. The right mastoid was used as the reference electrode and Ground was placed at AFZ. Impedances were reduced to below 8 kΩ. EEG was recorded continuously, digitized at 500 Hz and band-pass filtered (0.1 Hz to 75 Hz, including a 50 Hz notch filter). Processing was done off-line. The MMN and LDN are fronto-central responses and hence the data recorded at the relevant electrodes (F3, FZ, F4, C3, CZ, and C4) were extracted and submitted to further analysis.

### Offline analysis

Recordings of the responses to the three sets of stimuli were concatenated, and re-referenced to all electrodes before removing artefacts due to eye-blinks [55]. This method of re-referencing is effective in removing noise affecting many channels when the focus is on group differences rather than on the topography of responses.

After re-referencing, the continuous file was epoched from −100 to 1300 ms relative to stimulus onset and baseline corrected (−100 to 0 ms). EEG activity greater than ±120 µV was excluded from further analysis before calculating the averaged auditory ERP for each participant. Early analyses suggested that use of this criterion would maximise the number of trials per subject available for grand-averaging, thus improving the signal-to-noise ratio, while not having a detrimental effect on the final ERPs. Of a possible total of 618 standards and 144 deviants, 591 (s.d. 37) responses to standards in the good nonword-repeaters and 584 (s.d. 75) in the poor nonword-repeaters remained for grand averaging, while 138±1 responses were available for grand averaging for each deviant ERP for each group of nonword-repeater (s.d.s ranged from 8–17 across the two groups with no bias to greater or smaller s.d.s for either group). The averages were filtered using a 30 Hz low pass Finite Impulse Response filter, zerophase shift, 96 dB roll off.

Difference waves (Deviant minus Standard) were then calculated for each consonant change. A baseline of 80 ms prior to the onset of each syllable was used to correct for the impact of the response to the preceding syllable. Correction was performed by subtracting the average amplitude of this baseline from all data points in the analysis window. This is a conservative procedure that leads to reduced effect sizes compared to analysis of the uncorrected difference waves.

### Analysis of the difference waves

T-tests were performed at each point on the difference wave for all 59 participants to determine regions where the mean differed significantly from zero for at least 12 consecutive data points [56]. Two windows of mismatch response following each deviant were identified. The first occurred in the standard mismatch negativity (MMN) window around 100–200 ms after the onset of the consonant change and the second occurred around 200–600 ms after the consonant change. We refer to this latter response as the late discriminative negativity (LDN) [57].

Significant intervals differed only slightly from electrode to electrode and deviant to deviant, so a window was defined for MMN (82 to 218 ms from syllable onset) and LDN (226 to 630 ms from syllable onset) to encompass the whole range of significant regions across electrodes and deviants. The peak negativity was found on the difference wave in the MMN and LDN intervals for each Participant × Electrode × Deviant. Mismatch responses were defined using a similar approach to Čeponienė et al. [23]; taking MMN as the average amplitude over a 40 ms window centred on the peak and LDN as average amplitude over an 80 ms window centred on the peak. Averaging around an individual's peak response is preferable to taking mean amplitudes across the same window for all measurements when, as in this case, the window is wide and the peak mismatch response might be expected to show variable latency from one individual to another.

### Statistical analysis

A series of repeated-measures ANOVAs were performed comparing amplitude of MMN and LDN for the two groups with Electrode and Deviant position as the repeated measures. Multivariate test statistics are reported to avoid problems due to violations of sphericity. Alpha was set to 0.025 to maintain the family-wise type I error rate. Effect sizes are reported as η^2^. Where group main effects or interactions were significant, a further analysis was conducted to check whether the unequal group size between good and poor nonword-repeaters could lead to spurious findings. The good nonword-repeater group was randomly split into three subgroups, each with 14 or 15 cases. The analysis was re-run with all four groups to check whether the same effect was obtained. Planned comparisons were then used to test for the predicted difference between the poor nonword-repeater group and the other three groups combined.
